# *Drep1*, a Potential Mediator of miR-137, Modulates Yorkie-Driven Overgrowth in *Drosophila*

**DOI:** 10.3390/ijms27135718

**Published:** 2026-06-24

**Authors:** So-Min An, Kihan Tak, Jae-Yoon Yang, Dong-Seok Lee, Younghwi Kwon, Eunbyul Yeom

**Affiliations:** 1 BK21 FOUR KNU Creative BioResearch Group, School of Life Sciences, Kyungpook National University, Daegu 41566, Republic of Korea; algs1023@naver.com (S.-M.A.); tkh9296@naver.com (K.T.); jyyang324@naver.com (J.-Y.Y.); lee1@knu.ac.kr (D.-S.L.); 2 KNU-G LAMP Research Center, KNU-Institute of Basic Sciences, School of Life Sciences, College of Natural Sciences, Kyungpook National University, Daegu 41556, Republic of Korea

**Keywords:** hippo signaling, Yorkie (Yki), miRNA-137, Drep1, eye overgrowth, *Drosophila*

## Abstract

The Hippo–Yorkie (Yki) signaling pathway is a conserved regulator of tissue growth, and its dysregulation leads to excessive growth and tumorigenesis. Although several microRNAs (miRNAs) have been implicated in Hippo pathway regulation, how they modulate Yki activity in vivo remains incompletely understood. Here, we identify miR-137 as a suppressor of Yki-driven overgrowth in a *Drosophila* model. A functional miRNA screen revealed that miR-137 overexpression markedly suppresses Yki-induced eye overgrowth, whereas inhibition of miR-137 enhances eye overgrowth phenotypes. Through bioinformatic prediction and genetic screening, we identified Drep1 as a candidate downstream factor associated with miR-137 function. RNAi-mediated depletion of *Drep1* phenocopies the suppressive effects of miR-137, whereas *Drep1* overexpression enhances Yki-driven tissue overgrowth and proliferation. Consistent with these phenotypes, miR-137 overexpression or *Drep1* depletion reduces the expression of canonical Yki target genes, including *Diap1* and *Expanded*, indicating decreased Yki transcriptional output. Importantly, *Drep1* knockdown was associated with reduced Yki immunostaining in a complementary wing-disk context, suggesting a potential link between Drep1 and Yki-associated signaling. Consistent with this, miR-137 also reduced the expression of ICAD, the mammalian homolog of *Drep1*, providing preliminary evidence that miR-137 may regulate ICAD expression in mammalian cells. Together, these findings support a potential regulatory relationship between miR-137 and Drep1 that modulates Yki-driven eye overgrowth and reveal an additional layer of Hippo pathway regulation in vivo.

## 1. Introduction

The Hippo signaling pathway was originally identified in *Drosophila melanogaster* as a central regulator of organ size and tissue growth through the coordinated control of cell proliferation and apoptosis [1,2]. By integrating diverse upstream cues, including cell density, polarity, and mechanical inputs, Hippo signaling maintains tissue homeostasis and prevents excessive growth [3,4,5]. Disruption of this pathway leads to uncontrolled cell proliferation and tumorigenesis, underscoring its critical role in growth control [6,7,8].

In *Drosophila*, the Hippo pathway functions through a conserved kinase cascade composed of the serine/threonine kinase Hippo (Hpo) [2,9,10,11], the downstream kinase Warts (Wts) [12,13], and the adaptor proteins Salvador (Sav) [14,15] and Mats [16,17]. This kinase module phosphorylates and inhibits the transcriptional co-activator Yorkie (Yki), the principal downstream effector of the pathway [6,13,18]. When Hippo signaling is active, phosphorylated Yki is retained in the cytoplasm through interaction with 14-3-3 proteins, thereby preventing the transcription of genes that promote proliferation and survival [3,6,18]. Conversely, when Hippo signaling is attenuated, Yki translocates into the nucleus and cooperates with the transcription factor Scalloped (Sd) to induce target genes such as *Cyclin E*, *Diap1*, and *expanded*, driving tissue overgrowth and tumorigenesis [19,20].

The Hippo pathway is evolutionarily conserved [8,21]. In mammals, MST1/2 and LATS1/2 correspond to *Drosophila* Hpo and Wts, respectively, and regulate the Yki homologs YAP and TAZ [3,21]. Dysregulation of Hippo–YAP/TAZ signaling has been implicated in diverse human cancers, including liver, lung, breast, and colorectal cancers [3,22,23]. Owing to this conservation, *Drosophila* provides a powerful in vivo model to dissect mechanisms that modulate Hippo-driven tumorigenesis.

MicroRNAs (miRNAs) are small non-coding RNAs that post-transcriptionally regulate gene expression by targeting complementary sequences in mRNAs, leading to translational repression or transcript destabilization [24,25,26]. Through this mechanism, miRNAs fine-tune key biological processes, including proliferation, apoptosis, and tumor suppression [26]. Accumulating evidence indicates that miRNAs intersect with Hippo signaling at multiple levels [27]. In *Drosophila*, the Yki-induced miRNA *bantam* promotes tissue growth downstream of Yki [28,29], whereas in mammalian systems, miRNAs such as miR-375 and miR-582 suppress YAP/TAZ activity [30,31]. Despite these findings, how individual miRNAs modulate Yki activity in vivo—particularly in tumorigenic contexts—remains incompletely understood.

Here, we performed a functional miRNA-based genetic screen using a *Drosophila* Yki-driven overgrowth model and identified microRNA-137 (miR-137) as a potent suppressor of Yki-induced tissue overgrowth. Through genetic and molecular analyses, we identified Drep1 as a candidate miR-137-associated modifier that influences Yki-driven overgrowth. We further found that *Drep1* depletion is associated with reduced Yki immunostaining and altered Yki target-gene output in complementary epithelial contexts. These findings suggest a potential regulatory relationship among miR-137, Drep1, and Yki-driven overgrowth in vivo.

## 2. Results

### 2.1. miR-137 Suppresses Yki-Driven Eye Overgrowth

**Figure 1:**
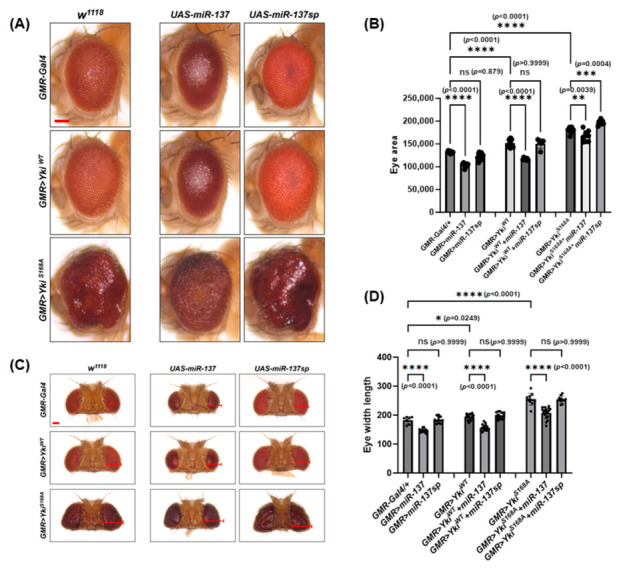
miR-137 suppresses Yorkie-driven eye overgrowth in *Drosophila*. (**A**) Representative images of female adult *Drosophila* eyes showing the effects of miR-137 overexpression or inhibition on Yki-driven eye overgrowth phenotypes. (**B**) Quantification of eye area corresponding to the phenotypes shown in (**A**). From left to right: *n* = 8, 8, 13, 16, 7, 5, 11, 8, 7. (**C**) Representative top-view images of adult fly heads showing eye phenotypes under the same genetic conditions as in (**A**). Reduced eye width was observed upon miR-137 overexpression in Yki-driven overgrowth. (**D**) Quantification of eye width corresponding to the phenotypes shown in (**C**). From left to right: *n* = 7, 14, 12, 16, 14, 14, 10, 18, 11. For all panels, data are presented as mean ± SD. Statistical significance was determined using One-way ANOVA with Tukey’s multiple comparison test (* *p* < 0.05, ** *p* < 0.01, *** *p* < 0.001, **** *p* < 0.0001, ns, not significant). Scale bars: 200 µm. The double-headed arrow indicates the eye width used for quantification.

To identify microRNAs (miRNAs) that modulate Yki-driven eye overgrowth, we performed a functional miRNA screen using a *Drosophila* eye overgrowth model. Eye-specific Yki-driven eye overgrowth models were generated using the *GMR-Gal4* driver. Two Yki variants were used: wild-type Yki (Yki^WT^) and a constitutively active mutant (Yki^S168A^), in which serine 168 is replaced by alanine to prevent Hippo-mediated inhibitory phosphorylation. Expression of Yki^WT^ (*GMR > Yki^WT^*) produced a mild rough-eye phenotype, whereas expression of Yki^S168A^ (*GMR > Yki^S168A^*) caused pronounced eye overgrowth accompanied by increased proliferative activity and elevated cell death (Figure 1A).

Using these models, we screened 145 miRNA lines for modifiers of Yki-induced eye phenotypes (Supplementary Figure S1, Supplementary Table S1), by evaluating cancer-relevant readouts, including tissue overgrowth, cell proliferation, and cell death. From the large-scale screen, six miRNAs were identified as candidate regulators of Yki-driven overgrowth: miR-277 and miR-911 (enhancers), and miR-137, miR-263a, miR-978, and miR-989 (suppressors) (Supplementary Figure S2A). To further validate the six candidate miRNAs identified in the screen, we performed an additional functional assay using the corresponding miRNA sponge lines (Supplementary Figure S2B). miRNA sponges are artificial RNA constructs that contain multiple binding sites complementary to the target miRNAs, thereby sequestering and inhibiting their activity. Therefore, we focused on selecting miRNAs that exhibit contrasting phenotypes when targeted by their respective functional sponges. Based on these results, four miRNAs (miR-137, miR-263a, miR-978, and miR-989) were selected as candidates. We selected miR-137 as our primary target based on previous findings demonstrating its inhibitory role in *Drosophila* eye development [32]. We hypothesized that this anti-proliferative effect could potentially mitigate the excessive tissue growth driven by the Hippo-Yki pathway dysregulation. Overexpression of miR-137 markedly suppressed Yki^S168A^-driven eye overgrowth and restored adult eye morphology toward control, whereas inhibition of miR-137 using a miR-137 sponge enhanced Yki-induced overgrowth (Figure 1A,B). Consistently, top-view imaging of adult fly heads revealed that miR-137 overexpression reduced eye width in the Yki-driven eye overgrowth model (Figure 1C,D). Together, these data establish miR-137 as a suppressor of Yki-driven eye overgrowth in *Drosophila*, supporting a functional role for miR-137 in Hippo–Yki-dependent overgrowth.

### 2.2. Drep1 Is Identified as a Candidate miR-137-Associated Modifier by Genetic Screening

**Figure 2:**
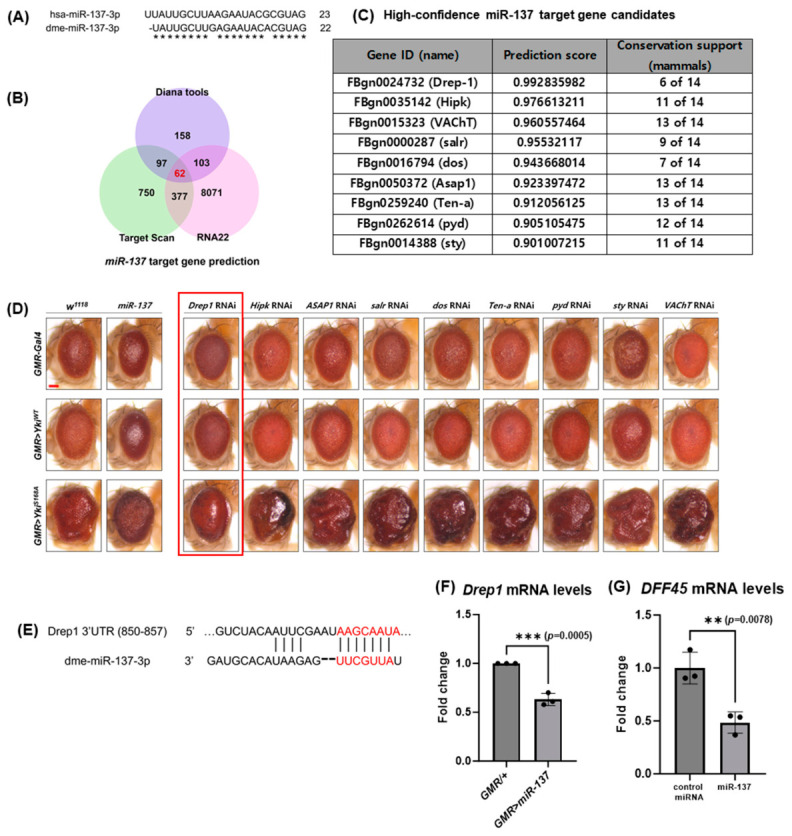
*Drep1* is a candidate miR-137-associated modifier identified by genetic modifier screening. (**A**) Sequence alignment of the mature *Drosophila* miR-137 (dme-miR-137-3p) and human miR-137 (hsa-miR-137-3p) showing strong conservation within the seed region. This conserved miR-137 sequence was used as the basis for subsequent target gene prediction analyses. Asterisks (*) indicate matching nucleotides between the aligned sequences. (**B**) Venn diagram summarizing the prediction of putative *miR-137* target genes using three computational tools: TargetScan, DIANA-microT-CDS, and RNA22. A total of 62 genes, including *Drep1*, were commonly predicted by all three tools. (**C**) Predicted high-confidence miR-137 target gene candidates ranked by prediction score. (**D**) Representative images of adult *Drosophila* eyes showing phenotypes resulting from RNAi-mediated knockdown of predicted miR-137 target genes in the *GMR-Gal4*-driven Yki eye overgrowth model. Knockdown of *Drep1* suppressed the Yki-induced overgrowth, which is highlighted with a red box. *n* = 5 flies per genotype. Scale bar: 200 µm. (**E**) Schematic representation of the predicted binding site for *dme-miR-137-3p* within the 3′ UTR of the *Drep1* mRNA. The complementary seed-matching sequence is highlighted in red. (**F**) Quantitative RT-PCR analysis of *Drep1* mRNA levels in control flies (*GMR-Gal4/+*) and flies overexpressing miR-137 under the control of *GMR-Gal4* (*GMR > miR-137*). Data are presented as mean ± SD from independent experiments. Biological replicate *n* = 3 (25 flies per group). (**G**) Quantitative RT-PCR analysis of *ICAD* (*DFF45*) mRNA levels in HEK293T cells transfected with control or miR-137 mimic. Data are presented as mean ± SD. Biological replicate *n* = 3. Statistical significance was determined using an unpaired Student’s *t*-test. ** *p* < 0.01, *** *p* < 0.001.

miR-137 is evolutionarily conserved from *Drosophila* to mammals, with strong conservation in its seed sequence (Figure 2A). Based on this conserved sequence, we performed target prediction using multiple algorithms and prioritized candidate targets supported across platforms and by mammalian conservation (Figure 2B). Out of 62 total candidates, nine candidates exhibiting a prediction score above 0.9 and high evolutionary conservation with their mammalian orthologs were selected for functional testing (Figure 2B,C and Supplementary Table S2) and evaluated by RNAi-mediated knockdown in the Yki eye overgrowth model to assess their ability to modify Yki-induced overgrowth. Among the candidates tested, knockdown of Drep1 markedly suppressed Yki-induced eye overgrowth, phenocopying the effect of miR-137 overexpression, whereas knockdown of other predicted targets did not produce comparable suppression in the Yki-driven overgrowth model (Figure 2D). Sequence analysis further identified a conserved miR-137 seed-matching site within the 3′ untranslated region (3′ UTR) of Drep1 (Figure 2E). To further examine whether Drep1 expression is associated with miR-137 activity in vivo, we overexpressed miR-137 in the eye using *GMR-Gal4* and quantified *Drep1* transcript levels. Quantitative RT-PCR revealed a significant reduction in *Drep1* mRNA upon miR-137 overexpression compared with controls (Figure 2F). Together, these results suggest that Drep1 is a candidate miR-137-associated modifier that may contribute to Yki-driven eye overgrowth. To explore the potential for a conserved regulatory relationship between miR-137 and Drep1, we examined the potential effect of miR-137 in a mammalian cell line. The *Drosophila* Drep1 protein is homologous to mammalian ICAD (also known as DFF45). In HEK293T cells, transfection of a miR-137 mimic was associated with a reduction in ICAD mRNA levels compared with control cells (Figure 2G). These findings provide a preliminary indication that miR-137 might be involved in the modulation of ICAD expression within a mammalian context. Although these findings do not establish direct targeting, they provide preliminary evidence that miR-137 may regulate ICAD expression in mammalian cells, suggesting the possibility of partial conservation of the possible miR-137-Drep1/ICAD regulatory relationship.

### 2.3. Drep1 Promotes Yki-Driven Eye Overgrowth and Proliferation

**Figure 3:**
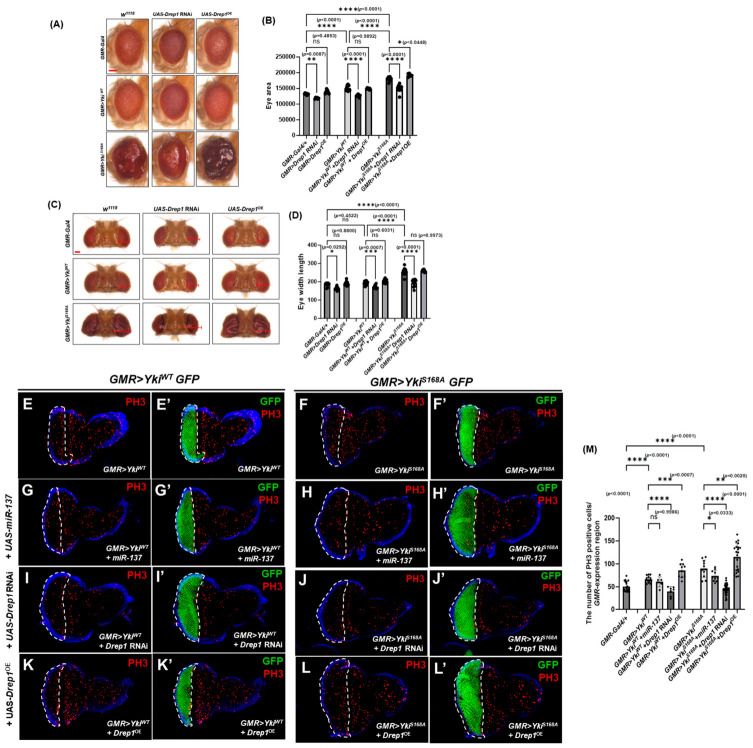
*Drep1* knockdown suppresses Yki-driven overgrowth and reduces proliferation in the *Drosophila* eye overgrowth model. (**A**) Representative images of adult *Drosophila* eyes showing the effects of manipulating the miR-137 and Drep1 on Yki-driven eye overgrowth phenotypes. Scale bar: 200 µm. (**B**) Quantification of adult eye area corresponding to the phenotypes shown in (**A**). Data are presented as mean ± SD. From left to right: *n* = 8, 6, 7, 15, 7, 7, 11, 12, 5. (**C**) Representative top-view images of adult fly heads showing eye phenotypes under the same genetic conditions as in (**A**). Scale bar: 200 µm. The double-headed arrow indicates the eye width used for quantification. (**D**) Quantification of eye width corresponding to the phenotypes shown in (**C**). Data are presented as mean ± SD. From left to right: *n* = 7, 14, 14, 16, 10, 16, 10, 13, 9. (**E**–**L′**) Confocal fluorescence microscopy images of third-instar larval eye-antennal disks immunostained for the mitotic marker phospho-histone H3 (PH3, red). Nuclei were counterstained with DAPI (blue). The dashed box indicates the GMR-expressing region used for quantification. Scale bar: 100 µm. (**M**) Quantification of PH3-positive mitotic cells within the Yki-expressing domain or the equivalent region of control disks corresponding to the genotypes shown in (**E**–**L′**). Data are presented as mean ± SD. From left to right: *n* = 19, 13, 6, 7, 10, 11, 10, 33, 21. Statistical significance was determined using One-way ANOVA with Tukey’s multiple comparison test (* *p* < 0.05, ** *p* < 0.01, *** *p* < 0.001, **** *p* < 0.0001, ns, not significant).

To determine whether Drep1 functionally contributes to Yki-driven overgrowth, we examined the effects of Drep1 knockdown and overexpression in the *Drosophila* eye overgrowth model. To confirm the efficiency of the *Drep1* RNAi line used in this study, we measured the Drep1 transcript levels by qRT-PCR. *Drep1* mRNA levels were significantly reduced in the *Drep1* RNAi line, confirming effective knockdown of *Drep1* (Supplementary Figure S3). RNAi-mediated knockdown of *Drep1* strongly suppressed Yki-induced eye overgrowth, whereas *Drep1* overexpression further enhanced Yki-driven eye enlargement (Figure 3A,B). Consistently, top-view imaging of adult fly heads showed that *Drep1* knockdown reduced, whereas *Drep1* overexpression increased eye width in the Yki-driven overgrowth model (Figure 3C,D). To test whether miR-137 and Drep1 modulate proliferative activity within Yki-induced overgrowth tissue, we assessed mitosis in larval eye–antennal disks by immunostaining for phospho-histone H3 (PH3). Overexpression of Yki significantly increased the number of PH3-positive mitotic cells within the Yki-expressing region, indicating elevated proliferative activity (Figure 3E,E′,F,F′,M). Consistent with the adult eye phenotypes, miR-137 overexpression significantly reduced PH3-positive cells in the Yki-driven overgrowth context (Figure 3G,G′,H,H′,M). Similarly, *Drep1* knockdown decreased PH3-positive cells in Yki-expressing disks, restoring mitotic activity toward control levels (Figure 3I,I′,J,J′,M and Supplementary Figure S4). To address whether this growth suppression is related to apoptosis, as Drep1 belongs to the apoptosis-associated ICAD family, we examined cleaved caspase-3 (CC3) levels. Notably, Drep1 depletion did not induce an obvious increase in CC3-positive signals in either Yki^WT^ or YkiS^168A^ overexpression contexts (Supplementary Figure S5), suggesting that Drep1 depletion suppresses Yki-driven overgrowth without detectable induction of apoptosis. In contrast, *Drep1* overexpression further increased PH3-positive cells within Yki-driven overgrowth tissue (Figure 3K,K′,L,L′,M). Together, these results indicate that Drep1 positively regulates Yki-driven overgrowth and proliferation, and that *Drep1* depletion is sufficient to attenuate Yki-induced overgrowth phenotypes in vivo.

To evaluate whether Drep1 also regulates tissue growth outside the Yki-driven overgrowth context, we examined the effects of Drep1 manipulation during normal eye and wing development (Supplementary Figures S6 and S7). *Drep1* knockdown driven by *ey-Gal4* caused severe growth suppression in the adult eye, indicating that Drep1 is required for proper eye development and that early depletion leads to excessive growth inhibition (Supplementary Figure S6A,B). In the wing, overexpression of *Drep1* using *en-Gal4* resulted in enlarged posterior wing, whereas knockdown of *Drep1* caused severe developmental defects that led to lethality (Supplementary Figure S7A,B). Consistently, PH3 analysis in wing disks showed decreased mitotic activity upon *Drep1* knockdown and increased mitotic activity upon *Drep1* overexpression within the *en-Gal4* expressing region (Supplementary Figure S7C,D). Collectively, these results suggest that Drep1 contributes to tissue growth and proliferative output under both developmental and Yki-driven overgrowth conditions.

### 2.4. miR-137 Overexpression and Drep1 Knockdown Reduce Yki Target Gene Expression

**Figure 4:**
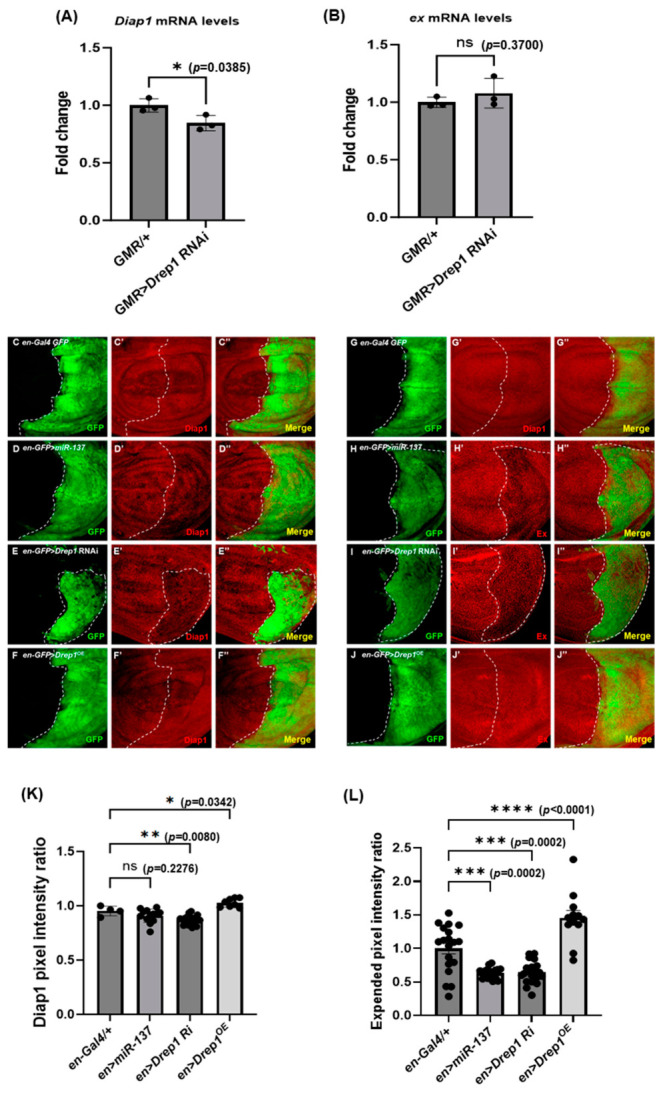
miR-137 overexpression and Drep1 knockdown reduce Yki target genes. Quantitative RT-PCR analysis of *diap1* (**A**) and *ex* (**B**) mRNA levels using *GMR-Gal4*-driven *Drep1* RNAi lines. Data are presented as mean ± SD from independent experiments. Biological replicate *n* = 3 (10 flies per group). Statistical significance was determined using an unpaired Student’s *t*-test. (**C**–**J″**) Confocal fluorescence microscopy images of third-instar larval wing disks immunostained for the Yki target gene Diap1 (red, Left) or Expanded (Ex, red, right). Control (*w^1118^*) (**C**–**C″**,**G**–**G″**), *UAS-miR-137* (**D**–**D″**, **H**–**H″**), *UAS-Drep1* RNAi (**E**–**E″**, **I**–**I″**), or *UAS-Drep1* overexpression (*Drep1*^OE^) (**F**–**F″**,**J**–**J″**) were expressed in the posterior compartment using the *en-Gal4* driver, with GFP (green) marking the region of expression. The dashed box indicates the GFP-positive region used for quantification. Scale bar: 50 µm. (**K**,**L**) Quantification of fluorescence intensity of Diap1 (**K**) and Expended (**L**) within *en-Gal4*-driven larval wing disk using Image J. From left to right: (**K**) *n* = 4, 13, 19, 8, (**L**) *n* = 19, 18, 20, 12. Data are presented as the mean ± SD. Statistical significance was determined using One-way ANOVA with Tukey’s multiple comparison test (* *p* < 0.05, ** *p* < 0.01, *** *p* < 0.001, **** *p* < 0.0001, ns, not significant).

Genetic interaction analyses suggested that miR-137 and *Drep1* influence Yki pathway output. To assess how miR-137 and *Drep1* modulate Yorkie pathway activity, we examined the expression of canonical Yki target genes in *GMR-Gal4*-driven eye disks, and we measured the transcript level of *Diap1* and *Expanded* (*Ex*). *Drep1* knockdown resulted in a reduction in *Diap1* transcript levels, whereas *Ex* levels were not significantly changed compared with controls (Figure 4A,B). These results suggest that *Drep1* depletion can affect at least a subset of Yki target-gene expression in the GMR-driven eye context, although individual Yki target genes may respond differently. Because the GMR-based qRT-PCR analysis provided limited target-gene coverage and did not fully resolve pathway-output changes at the tissue level, we next used the en-Gal4 larval wing-disk system as a complementary epithelial context to assess Yki target protein expression. Overexpression of *miR-137* markedly reduced Ex protein levels within the *en-GFP*-positive posterior compartment (Figure 4D–D″,H–H″). Similarly, RNAi-mediated knockdown of *Drep1* decreased *Diap1* and *Ex* expression, consistent with reduced Yki transcriptional output (Figure 4E–E″,I–I″). In contrast, Drep1 overexpression moderately increased both Diap1 and Ex levels (Figure 4F–F″,J–J″). Together, these results provide supportive evidence that Drep1 manipulation is associated with changes in Yki target-gene expression. However, because the GMR-based qRT-PCR analysis showed differential responses between Diap1 and ex, and the wing-disk assay represents a complementary epithelial context, these data should be interpreted as supportive rather than definitive evidence for altered Yki pathway output.

### 2.5. Drep1 Knockdown Is Associated with Reduced Yki Immunostaining in the Wing-Disk Context

**Figure 5:**
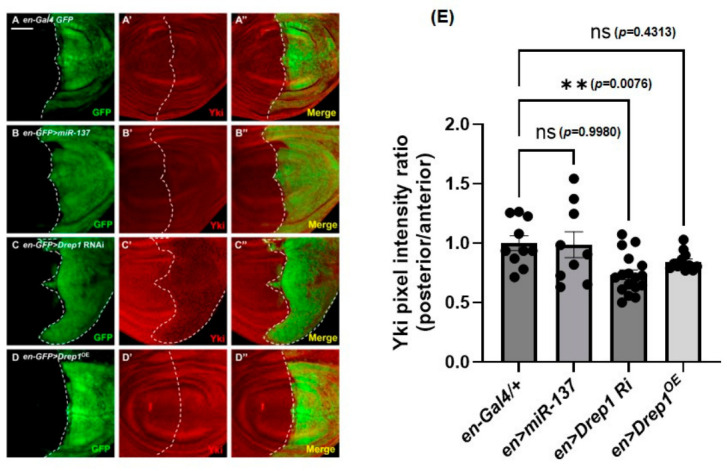
*Drep1* knockdown lowers Yki protein abundance without altering subcellular localization. Confocal fluorescence microscopy images of third-instar larval wing disks immunostained for Yki (red). Control wing disc (*en-Gal4*/*w^1118^*) (**A**–**A″**), *UAS-miR-137* (**B**–**B″**), *UAS-Drep1* RNAi (**C**–**C″**), or *UAS-Drep1* overexpression (*Drep1*^OE^) (**D**–**D″**) were expressed in the posterior compartment using the *en-Gal4* driver, with GFP marking the region of expression (green). The dashed box indicates the GFP-positive region used for quantification. Scale bar: 50 µm. (**E**) Quantification of fluorescence intensity of Yki within *en-Gal4*-driven larval wing disks using Image J. From left to right: *n* = 10, 9, 17, 10. Data are presented as the mean ± SD. Statistical significance was determined using one-way ANOVA with Tukey’s multiple comparison test (** *p* < 0.01, ns, not significant).

In the canonical Hippo pathway, Yki activity is regulated by phosphorylation-dependent cytoplasmic sequestration, and changes in Yki subcellular localization are widely used as a readout of Hippo signaling [6,18]. To test whether miR-137 and Drep1 regulate Yki at the level of protein abundance and/or localization, we examined Yki protein levels and subcellular distribution in larval tissues (Figure 5). Under these conditions, miR-137 overexpression alone did not robustly decrease Yki protein abundance (Figure 5B–B″,E). In contrast, *Drep1* knockdown consistently reduced total Yki protein levels, whereas *Drep1* caused no significant change in Yki protein abundance (Figure 5C–C″,D–D″,E), suggesting that Drep1 contributes to the maintenance of Yki protein abundance. Despite these changes in Yki protein abundance, Yki subcellular localization did not show detectable alterations upon miR-137 overexpression, *Drep1* knockdown, or *Drep1* overexpression (Supplementary Figure S8A–D′′′,E). In addition, RT-PCR analysis using *actinGS-Gal4 or GMR-Gal4* driver showed no significant change in Yki transcript levels under the corresponding genetic manipulation (Supplementary Figure S9). Although this analysis was not performed in a tissue-matched larval wing disk, the results suggest that Drep1-mediated regulation of Yki may occur primarily at the protein level. Together, these results suggest that Drep1 manipulation is associated with altered Yki immunostaining/protein abundance in the en-Gal4 wing-disk context. However, because these data were obtained in a complementary epithelial context and do not establish a direct protein-stability mechanism, further tissue-matched and biochemical analysis will be required to define how Drep1 influences Yki regulation.

## 3. Discussion

Our study identifies *Drep1* as a previously unrecognized regulator of Hippo–Yorkie signaling in Yki-driven overgrowth contexts in *Drosophila*. Through a functional miRNA screen using a Yki-induced eye overgrowth model, we identified miR-137 as a suppressor of Yki-driven tissue overgrowth and subsequently uncovered *Drep1* as a potential functional mediator downstream of miR-137. Genetic and molecular analyses demonstrated that depletion of *Drep1* phenocopies the suppressive effects of miR-137 and is accompanied by a pronounced reduction in Yki transcriptional output. Conversely, *Drep1* overexpression enhances Yki-driven tissue overgrowth and Yki target gene expression. Notably, *Drep1* depletion resulted in a marked reduction in Yki protein abundance without detectable changes in Yki subcellular localization or *yki* transcript levels, suggesting that the observed effects are unlikely to be primarily explained by altered Yki localization or detectable changes in *yki* transcript levels.

Together, these findings support a model in which Drep1 manipulation was associated with altered Yki immunostaining/protein abundance in the complementary wing-disk context. This suggests a post-transcriptional layer of regulation that may influence Yki protein stability or turnover [33]. While Hippo signaling is classically controlled through phosphorylation-dependent cytoplasmic sequestration, accumulating evidence indicates that additional mechanisms contribute to fine-tuning Yki/YAP activity [3,8]. Our data place Drep1 within this emerging regulatory layer and highlight protein abundance control as a potentially important mechanism in tumor contexts characterized by elevated Yki activity.

*Drep1* belongs to the DNA fragmentation factor (DFF) family and has been primarily studied in the context of apoptosis-associated nuclear events [34]. In *Drosophila*, *Drep1* forms a complex with the endonuclease *Drep4* and inhibits its nuclease activity under basal conditions, whereas caspase-mediated cleavage of Drep1 during apoptosis releases *Drep4* to promote DNA fragmentation and chromatin condensation [35,36]. This functional relationship parallels the mammalian ICAD–CAD system, in which ICAD functions as both a chaperone and inhibitor of CAD [37,38]. Although *Drep1* has traditionally been studied in the context of apoptosis, our findings extend its functional relevance by implicating *Drep1* in the regulation of tissue growth and Yki-driven eye overgrowth. These observations suggest an unexpected connection between apoptosis-related regulatory modules and Hippo–Yorkie signaling. Importantly, *Drep1* depletion did not induce obvious increases in cleaved caspase-3 signals under the Yki overgrowth conditions used in this study, suggesting that the suppression of Yki-driven overgrowth is not primarily attributable to widespread apoptotic cell death.

Our study further links Drep1 to the conserved eye overgrowth-suppressive miR-137. A recent study demonstrated that miR-137 suppresses *Drosophila* eye growth by repressing *Myc* and inducing caspase-dependent apoptosis, as evidenced by increased Dcp-1 activity [32]. However, our results show that Drep1 depletion effectively suppresses Yki-driven eye overgrowth without inducing any obvious increase in CC3-positive signals under either Yki^WT^ or Yki^S168A^ overexpression. These observations suggest that the suppressive effect of *Drep1* depletion on Yki-driven overgrowth is unlikely to be mediated by caspase-dependent apoptosis. While miR-137 is known to engage in an apoptotic pathway, our findings implicate Drep1 as a mediator of a caspase-independent mechanism. Together, these observations suggest that miR-137-*Myc* and miR-137-*Drep1* may function as parallel downstream pathways to regulate tissue growth. Consistent with this possibility, our finding implicates Drep1 as an additional potential mediator of miR-137 in Yki-driven overgrowth. In mammalian systems, miR-137 functions as a tumor suppressor in multiple cancer types and has been implicated in modulating YAP/TAZ signaling via upstream regulatory components [39,40]. In this context, our findings suggest that miR-137 constrains oncogenic growth through context-dependent downstream effectors and identify *Drep1* as a previously unappreciated mediator of miR-137 function in Yki-driven eye overgrowth.

Although Drep1 knockdown was associated with reduced Yki immunostaining in wing disks, the precise mechanism underlying this effect remains unresolved. In particular, strong *Drep1* depletion in the *en-Gal4* wing-disk context causes severe developmental defects, which may complicate transcriptional and protein-level interpretation. Therefore, we interpret the wing-disk Yki staining data as supportive evidence for an association between Drep1 manipulation and altered Yki immunostaining/protein abundance, but not as direct evidence for Yki protein stability or turnover. Further studies using tissue-matched genetic conditions, endogenous Yki reporters, and biochemical protein-turnover assays will be required to determine whether Drep1 directly regulates Yki abundance. In addition, although *Drep1* knockdown was validated by qRT-PCR, the use of a single RNAi line remains a limitation. Future independent RNAi or rescue experiments will be needed to further confirm reagent specificity.

Finally, given that miR-137 suppresses YAP/TAZ activity in human cancers and that ICAD/DFF45 is the mammalian homolog of *Drep1*, it will be important to investigate whether a conserved possible regulatory link between miR-137 and ICAD influences YAP/TAZ protein abundance in mammalian systems. Notably, we observed that miR-137 also reduces the expression of ICAD, the mammalian homolog of Drep1, in human cells. This finding provides preliminary evidence that miR-137 may regulate ICAD, although further studies will be required to determine whether this interaction is directly mediated through the predicted 3′UTR binding site. Future studies will be required to determine whether similar regulatory relationships operate in mammalian Hippo/YAP signaling contexts. In summary, our study identifies *Drep1* as a candidate modifier of Yki-driven overgrowth that is associated with altered Yki immunostaining/protein abundance in a complementary wing-disk context. By suggesting a potential association between miR-137, Drep1, and Yki-associated overgrowth in vivo, this study expands our understanding of how microRNAs and apoptosis-associated factors intersect with Hippo signaling to modulate oncogenic tissue overgrowth.

## 4. Materials and Methods

### 4.1. Drosophila Genetics

*Drosophila melanogaster* flies were maintained at 25 °C and normal humidity conditions (60%) on cornmeal, yeast, and molasses medium under identical rearing conditions. Unless otherwise stated, *w^1118^* flies were used as controls. The following *Drosophila* lines were used in this study: *w^1118^*, *GMR-Gal4*, *GMR > Yki^WT^*, *GMR > Yki^S168A^*, *eyeless-Gal4* (*ey-Gal4*), *engrailed-Gal4* (*en-Gal4*), *actinGS-Gal4*, microRNA sponge lines, and the microRNA overexpression lines used for microRNA screening, all kindly provided by Dr. Kweon Yu (KRIBB, Daejeon, Republic of Korea). RNA interference (RNAi) lines, including *UAS-Drep1* RNAi (BDSC, BL65944), were obtained from the Bloomington *Drosophila* Stock Center (Bloomington, IN, USA). Korea Drosophila Resource Center (KDRC) provided microinjection services to create the following transgenic *Drosophila* strain: UAS-Drep1. For the Gene Switch system using *actinGS-Gal4*, adult flies were fed on medium containing 200 µM mifepristone (RU486, Sigma-Aldrich, St. Louis, MO, USA; M8046) for 5 days before analysis, while control flies were fed on medium containing an equal volume of ethanol as a vehicle.

### 4.2. Imaging and Quantification of Eye and Wing Phenotypes

Adult flies aged for 3–5 days after eclosion were anesthetized and frozen at −20 °C for approximately 4 h prior to imaging of adult eyes. For eye swelling analysis, adult heads were dissected before imaging. For wing size analysis, adult wings were dissected from flies of the same age. All samples were imaged using an IMT camera (Olympus SZ61; Olympus SZ61; Olympus Corporation, Tokyo, Japan) under identical acquisition settings. Eye area was quantified from frontal eye images using ImageJ software 8 (National Institutes of Health, Bethesda, MD, USA). Eye width was measured as the linear distance from the midpoint of the head–eye boundary to the anterior margin of the eye using ImageJ. Wing area was quantified from dissected wing images using ImageJ.

### 4.3. Immunofluorescence Staining

Imaginal disks were dissected from third-instar larvae in phosphate-buffered saline (PBS) and fixed in 4% paraformaldehyde (4% PFA) for 30 min at room temperature. Following fixation, samples were washed three times with PBST (0.1% Triton X-100 in 1 × PBS) and blocked in 4% bovine serum albumin (BSA) in PBST for 30 min at room temperature. Samples were incubated overnight at 4 °C with the following primary antibodies: rabbit anti-phospho-histone H3 (PH3; 1:200; Cell Signaling Technology, Danvers, MA, USA; #9701S), mouse anti-Diap1 (1:100; gift from Dr. Bruce A. Hay), rat anti-Expanded (1:100; ABclonal, Wuhan, China; #WG-02450D), and rabbit anti-Yki (1:400; gift from Dr. Matthew Gibson, The University of Manchester, Manchester, UK). After washing the tissues five times with PBST, samples were incubated overnight at 4 °C with the following secondary antibodies: goat anti-rabbit IgG conjugated to Alexa Fluor 594 (1:500; BioActs, Incheon, Republic of Korea; #RSA1295), goat anti-rat IgG conjugated to Alexa Fluor 594 (1:500; BioActs, Incheon, Republic of Korea; #RSA1595), and goat anti-mouse IgG conjugated to Alexa Fluor 594 (1:500; BioActs, Incheon, Republic of Korea; #RSA1195). After washing the tissues five times with PBST, samples were mounted using a mounting solution (VECTASHIELD, vector Laboratories, Newark, CA, UAS; H-1200-10) and imaged using a confocal laser scanning microscope (LSM 800, Zeiss, Oberkochen, Germany). Regions of interest (ROIs) were defined and measured using identical parameters across all samples. All images within each experiment were acquired using identical microscope settings and analyzed using the same workflow.

### 4.4. Cell Culture and Transfection

HEK293T (Human embryonic kidney) cells were obtained from the American Type Culture Collection (ATCC, Manassas, VA, USA) and were maintained in Dulbecco’s modified Eagle’s medium (DMEM; Gibco, Thermo Fisher Scientific, Waltham, MA, USA) supplemented with 10% heat-inactivated fetal bovine serum (FBS; Gibco, Thermo Fisher Scientific, Waltham, MA, USA) and 50 µg/mL penicillin-streptomycin at 37 °C in a humidified atmosphere of 5% CO_2_. For miRNA transfection, cells were seeded in a 6-well plate (3 × 10^5^ cells/well) and were transfected with 50 nM of either control miRNA (Bioneer, Daejeon, Republic of Korea; AccuTarget^TM^ miRNA Negative Control) or miR-137 (Bioneer, Daejeon, Republic of Korea; hsa-miR-137 mimic, Accession MI0000454) using AccuFect^TM^ Transfect reagent (Bioneer, Daejeon, Republic of Korea; K-7920) following the manufacturer’s protocol for 48 h. After transfection, target genes were confirmed by quantitative RT-PCR analysis.

### 4.5. RNA Extraction and Quantitative RT-PCR

Total RNA was extracted from cells and flies using the easy-BLUE^TM^ Total RNA Extraction Kit (iNtron, Biothechnology, Seongnam, Republic of Korea; Cat.17061). Using extracted RNA, DNA was synthesized with the High-Capacity cDNA Reverse Transcription Kit of Applied biosystems (Applied Biosystemes^TM^, Foster City, CA, USA; #4368814). Quantitative RT-PCR was performed using Power SYBR^TM^ Green PCR Master Mix (Applied Biosystems^TM^, Foster City, CA, USA; 4367659). Relative mRNA expression levels were normalized to RP49 (fly) and 18s rRNA (cell). The 2^−ΔΔCt^ method was used to analyze relative changes in gene expression. Melt-curve analysis showed a single peak for all primer pairs. Primer sequences were as follows: RP49 (F: AGATCGTGAAGAAGCGCACCAAG, R: CACCAGGAACTTCTTGAATCCGG), Drep1 (F: TGGTGGACATCACGGGAAAG, R: TCAACTGCCGTCCAATCTCC), Yki (F: GAGCAGGCAGTTACCGAGTC, R: AAGACGGCGGATTTACAATG), Diap1 (F: CGCCTCCTCTGTGACAAAGT, R: TCACATCGGTGAAGGGCTTC), Ex (F: CGAGCATTTTGCACCGTCAG, R: GCATCGACTAGGAAGTACAAGGT), DFF45 (F: GCAGGGTTGAAGTGGAAGAATG, R: AGGAGGATGATGCTGGACAGAT), 18s rRNA (F: CGGACCAGAGCGAAAGCAT, R: CCTCCGACTTTCGTTCTTGATT). Primer specificity was confirmed by melt-curve analysis, showing a single peak for each primer pair. Primer efficiencies were checked or assumed to be comparable across primer sets under the experimental conditions used.

### 4.6. Statistical Analysis

Statistical analyses were performed using GraphPad Prism 9.5.1 software (GraphPad Software, San Diego, CA, USA). For comparisons between two experimental groups, a two-tailed unpaired Student’s *t*-test was employed. For multi-group comparisons, *p* values shown in the figures and legends represent Tukey-adjusted *p* values following one-way ANOVA. Significance levels were indicated as follows: **** *p* < 0.0001, *** *p* < 0.001, ** *p* < 0.01 and * *p* < 0.05 or n.s. (not significant).

## Data Availability

The original contributions presented in this study are included in the article/Supplementary Materials. Further inquiries can be directed to the corresponding author.

## Supplementary Materials

The following supporting information can be downloaded at: https://www.mdpi.com/article/10.3390/ijms27135718/s1.
