# Supplementary material for: Drep1, a Potential Mediator of miR-137, Modulates Yorkie-Driven Overgrowth in Drosophila
Source: Int J Mol Sci. 2026 Jun 24;27(13):5718. doi: 10.3390/ijms27135718 (PMC13362088; doi:10.3390/ijms27135718)
Supplement: Supplementary file 1 [file ijms-27-05718-s001.zip › Supplementary Figures and legends final.pdf]

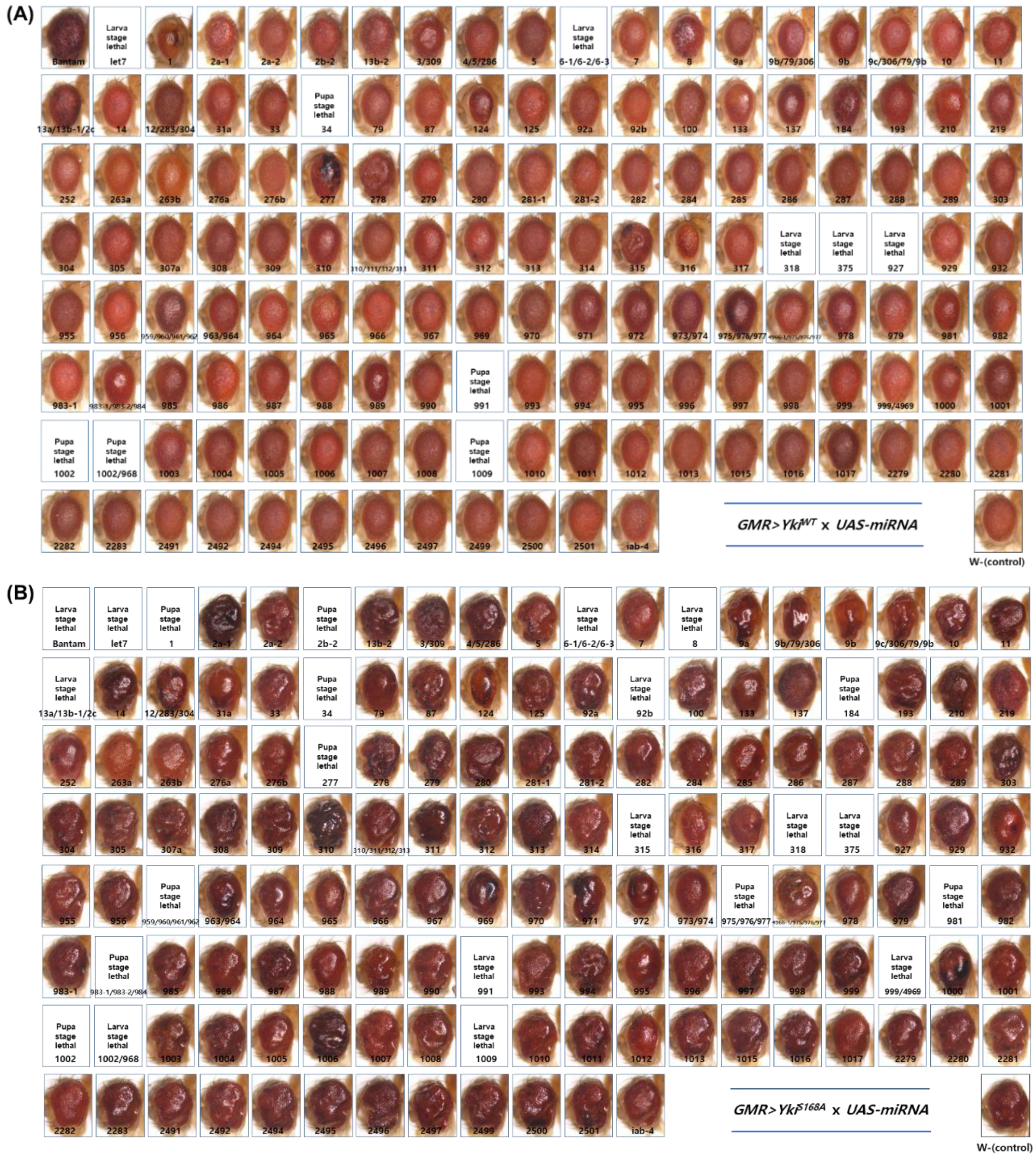

Supplementary Figure S1. microRNA (miR) screening using *Drosophila* Yki-driven eye overgrowth model expressing UAS-Yki^{WT} (A) and UAS-Yki^{S168A} in the eye.

Screening of miRNAs(miRs) using *Drosophila* Yki-driven eye overgrowth model. *UAS-Yki^{WT}* (A) or *UAS-Yki^{S168A}* (B) was expressed in the eye using *GMR-Gal4*, and these flies were crossed with *UAS-miRNA* library lines to examine modifier effects on eye phenotypes. Control flies, *GMR>Yki^{WT}*/+ (A) and *GMR>Yki^{S168A}*/+ (B) are shown in the bottom-right panels. *n*=5 female flies per group.

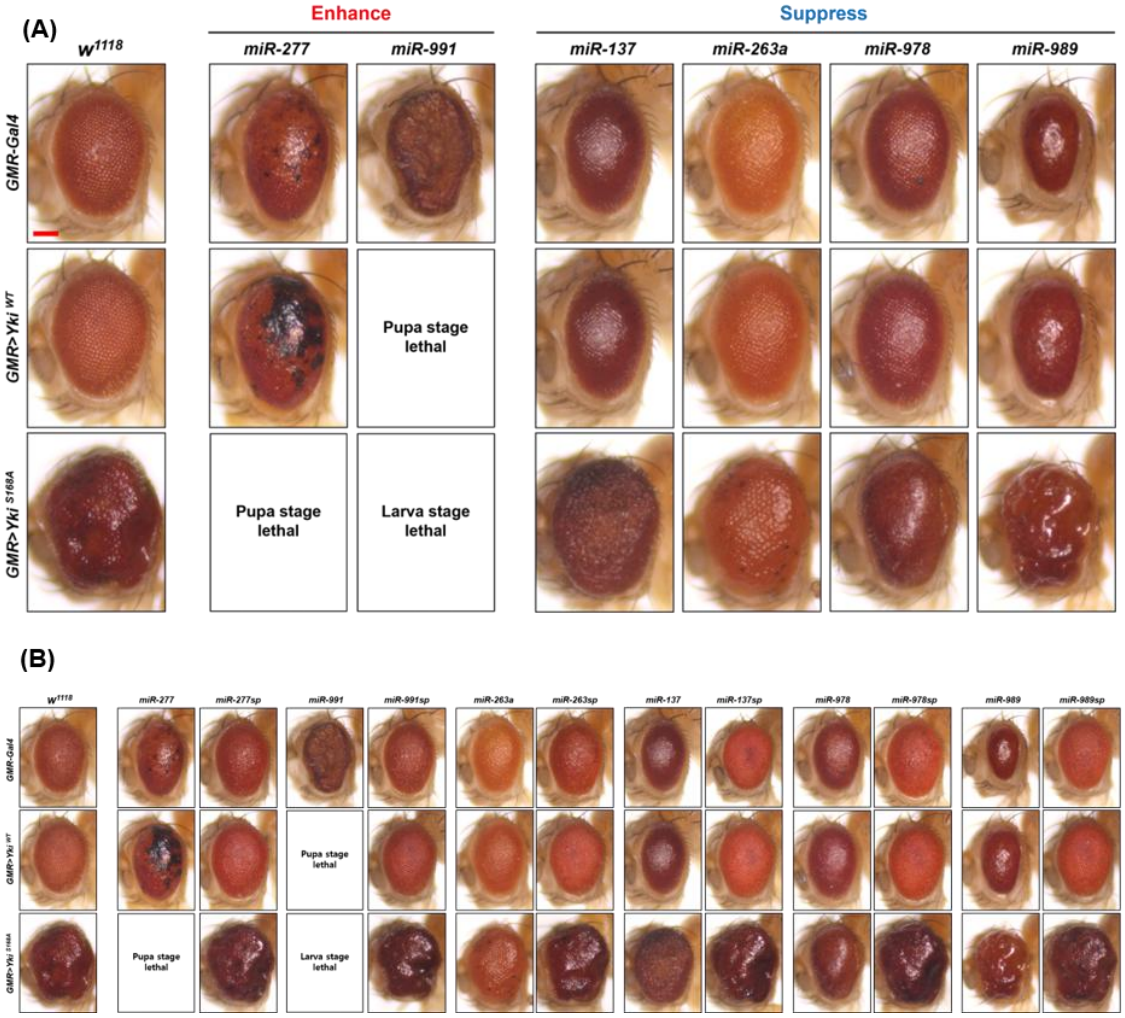

Supplementary Figure S2. Identification of six candidate microRNAs modulating Yki-driven eye overgrowth phenotypes in *Drosophila*.

(A) Representative images of *Drosophila* eyes showing six candidate miRNAs that modified Yki-driven eye overgrowth phenotypes. Among these candidates, miR-277 and miR-911 enhanced Yki-induced overgrowth, whereas miR-137, miR-263a, miR-978, and miR-989 suppressed Yki-driven eye overgrowth. n = 5 female flies per group. (B) Representative images of *Drosophila* eyes expressing UAS-miRNA and miRNA sponge lines for each candidate under the control of *GMR-Gal4*. Sponge lines were also co-expressed with *GMR>Yki*^{WT} or *GMR>Yki*^{S168A} to assess modifier effects on Yki-driven eye overgrowth. n = 5 female flies per group.

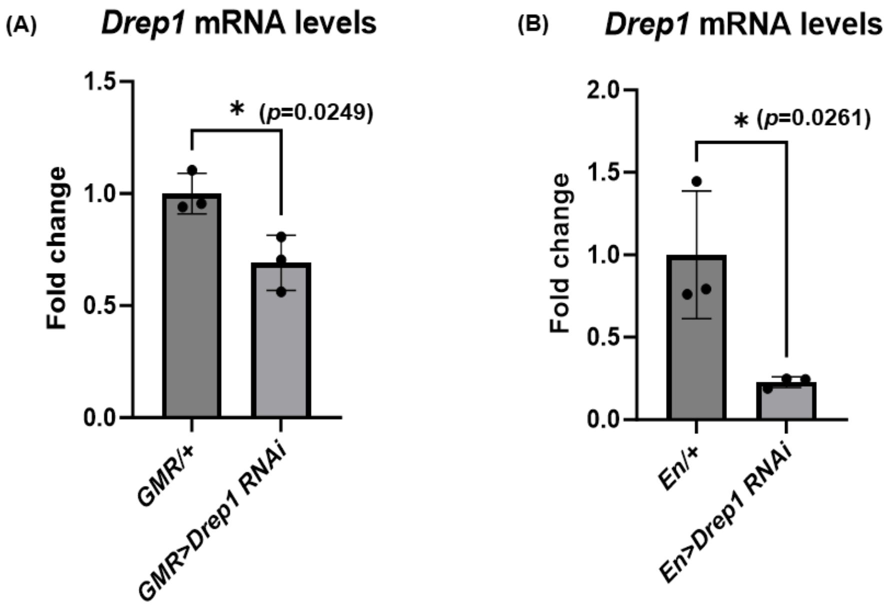

Supplementary Figure S3. Validation of *Drep1* knockdown efficiency.

Quantitative RT-PCR analysis of *Drep1* mRNA levels using *GMR-Gal4*-driven (A) and *en-Gal4*-driven (B) *Drep1* RNAi samples. *Drep1* RNAi significantly reduced *Drep1* transcript levels compared with the corresponding controls. Data are presented as mean ± SD from independent experiments. Biological replicate n = 3; 10 flies per biological replicate. Statistical significance was determined using an unpaired Student's t-test.

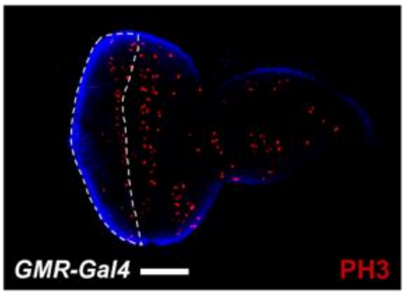

Supplementary Figure S4. The PH3 staining in *GMR-Gal4* eye discs.

Confocal fluorescence image of a third-instar larval eye–antennal disc expressing *GMR-Gal4* alone, immunostained for phospho-histone H3 (PH3, red). Nuclei are counterstained with DAPI (blue). This control image illustrates basal mitotic activity in the *GMR-Gal4* domain used for comparison with Yki and miR-137/Drep1 manipulations. Scale bar: 100 μm.

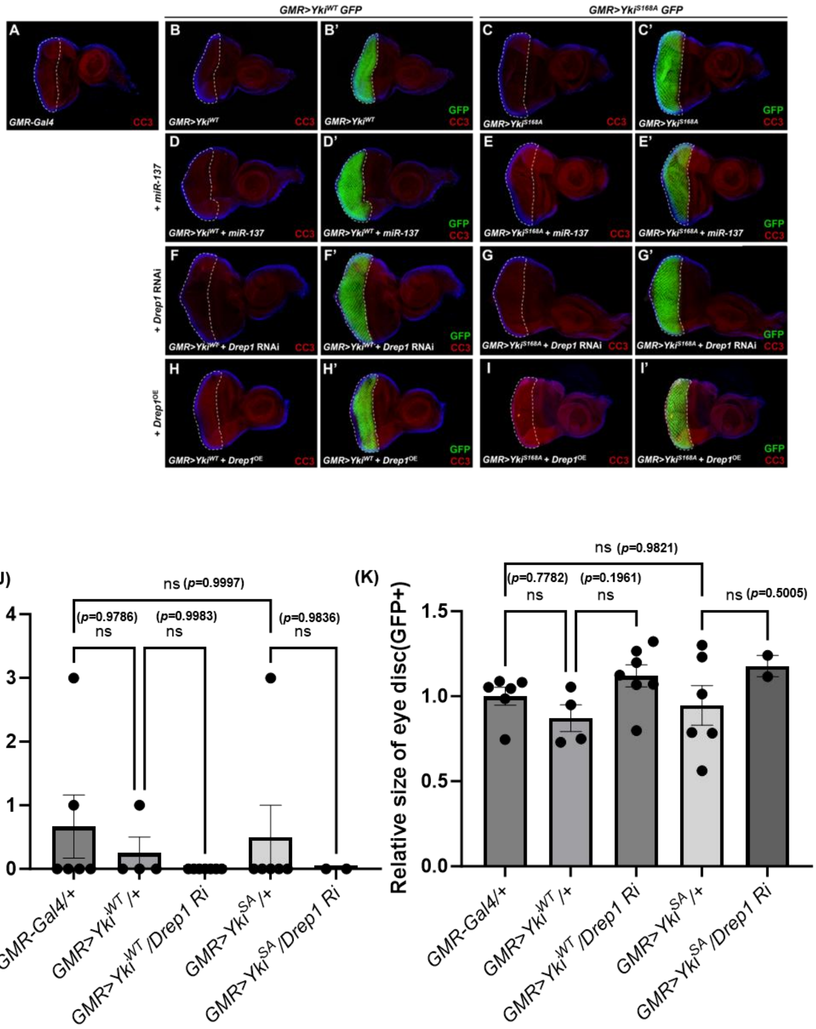

Supplementary Figure S5. Knockdown of *Drep1* does not alter cleaved caspase-3 (CC3) levels in Yki-driven overgrowth model.

(A-I') Representative image of third-instar larval eye-antennal discs immunostained for apoptotic marker cleaved caspase-3 (CC3,red). (A) Control eye-antennal disc (*GMR-Gal4/W^{1118}*) (A). Discs expressing Yki^{WT} alone (B-B") or co-expressed with *UAS-miR-137* (D-D"), *UAS-Drep1* RNAi (F-F"), *UAS-Drep1^{OE}* (H-H"). Discs expressing Yki^{S168A} alone (C-C") or co-expressed with *UAS-miR-137* (E-E"), *UAS-Drep1* RNAi (G-G"), *UAS-Drep1^{OE}* (I-I"). Scale bar: 100μm. (J-K) Quantification of CC3-positive signal intensity (J) and GFP-positive domain area (K) within *GMR-Gal4*-driven larval eye–antennal discs using ImageJ. From left to right: n = 6, 4, 7, 6, and 2 discs per genotype. Data are presented as mean ± SD. Statistical significance was determined using one-way ANOVA with Tukey's multiple comparison test.

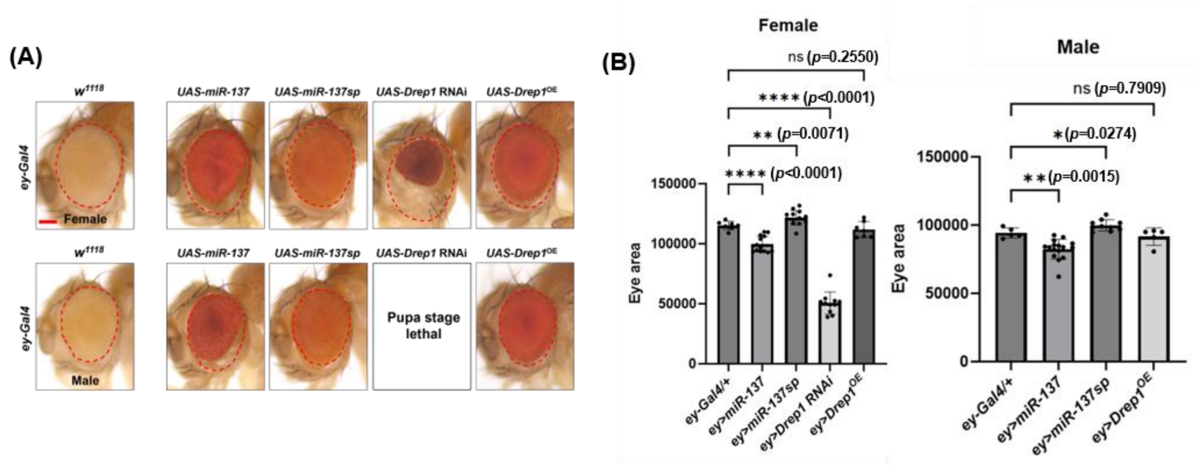

Supplementary Figure S6. miR-137 and Drep1 modulate eye development in *Drosophila.*

(A) Representative images of female and male adult *Drosophila* eyes showing phenotypes resulting from manipulating miR-137 and Drep1. Scale bar: 200 µm. (B) Quantification of eye area corresponding to the phenotypes shown in (A). Data are presented as mean ± SD. From left to right: female *n*=7, 12, 10, 10, 7, male *n*=5, 12, 8, 5. Statistical significance was determined using One-way ANOVA with Tukey's multiple comparison test.

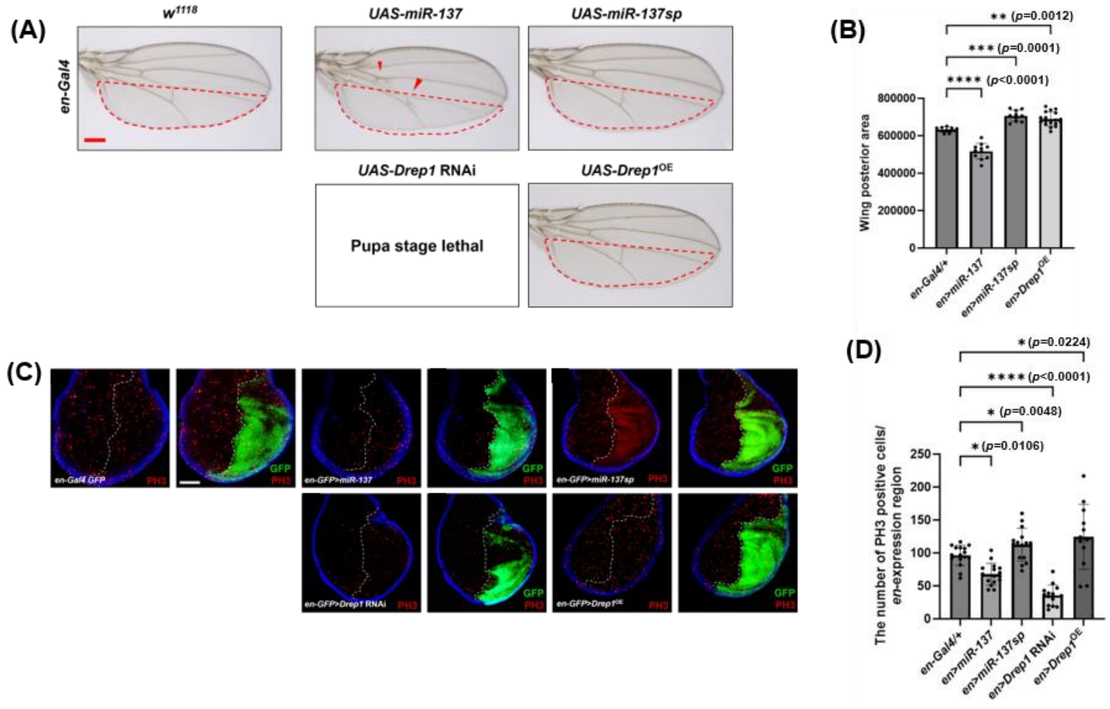

Supplementary Figure S7. miR-137 and Drep1 modulate wing development in *Drosophila.*

(A) Representative images of adult *Drosophila wings* showing phenotypes resulting from manipulating miR-137 and Drep1. Scale bar: 200 μm. (B) Quantification of wing area corresponding to the phenotypes shown in (A). Data are presented as mean ± SD. *n*=5 (*en-Gal4/+, en>miR-137, en>miR-137 sp*) and *n*=18 (*en>Drep1*^{OE}). Adult wings from *en>Drep1* RNAi flies were not available due to severe developmental defects and lethality. (C) Confocal fluorescence microscopy images of third-instar larval wing discs immunostained for the mitotic marker phospho-histone H3 (PH3, red). Scale bar: 50 μm. (D) Quantification of PH3-positive cells corresponding to the genotypes shown in (C). Data are presented as mean ± SD. *n*=12 wing discs per genotype. Statistical significance was determined using a One-way ANOVA with Tukey's multiple comparison test.

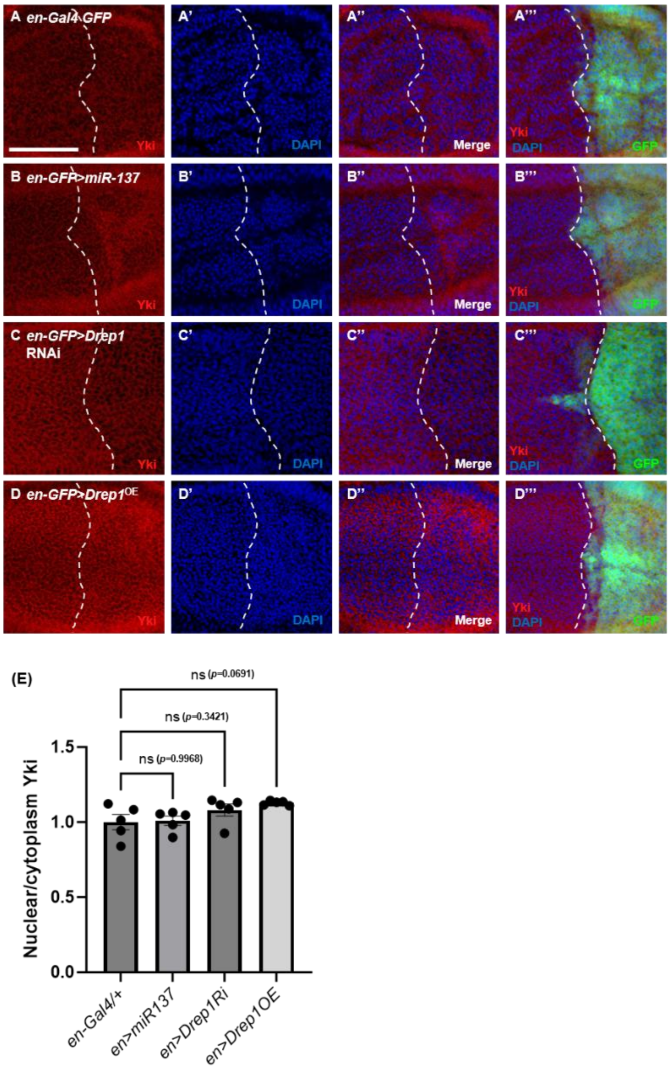

Supplementary Figure S8. Quantification of Yki nuclear/cytoplasmic distribution in the wing-disc context.

(A-D´´´) Confocal fluorescence microscopy images of third-instar larval wing discs immunostained for Yki (red). Nuclei were counterstained with DAPI (blue). UAS-miR-137, UAS-Drep1 RNAi, or UAS-Drep1 overexpression (Drep1OE) was expressed in the posterior compartment using en-Gal4, with GFP marking the region of expression. (E) Quantification of the nuclear-to-cytoplasmic ratio of Yki fluorescence intensity in *en-Gal4*-driven larval wing discs using ImageJ. n = 5 discs per genotype. Statistical significance was determined using one-way ANOVA with Tukey's multiple comparison test.

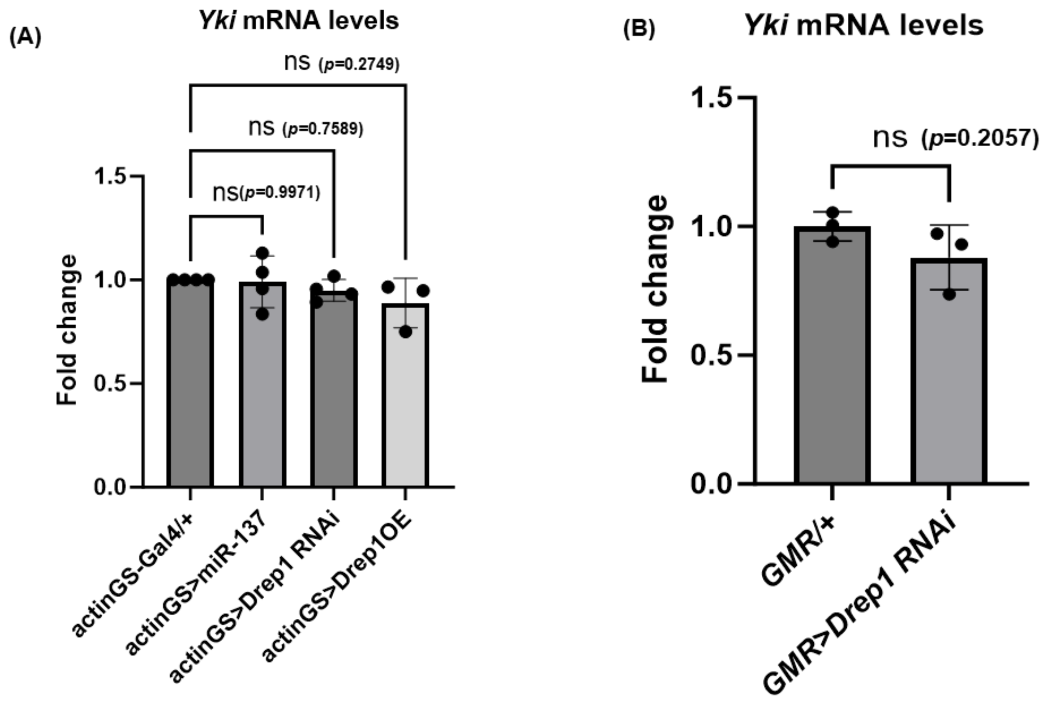

Supplementary Figure S9. Quantitative RT-PCR analysis of *yki* transcript levels.

(A) yki transcript levels in whole-body adult flies expressing UAS-miR-137, UAS-Drep1 RNAi, or UAS-Drep1OE under the control of *actinGS-Gal4*. Biological replicates: n = 4, 4, 4, and 3; 15 flies per biological replicate. (B) yki transcript levels in *GMR-Gal4*-driven eye samples comparing control and *GMR>Drep1 RNAi*. Biological replicate n = 3; 10 flies per biological replicate. Data are presented as mean ± SD. Statistical significance was determined using one-way ANOVA with Tukey's multiple comparison test (A) or an unpaired Student's t-test (B).
